# Metagenomics analysis yields assembled genomes from prokaryotic anaerobes with polymer‐degrading potential

**DOI:** 10.1002/btpr.3484

**Published:** 2024-06-17

**Authors:** Elaina M. Blair, Jennifer L. Brown, Dong Li, Patricia A. Holden, Michelle A. O'Malley

**Affiliations:** ^1^ Department of Chemical Engineering University of California Santa Barbara California USA; ^2^ Bren School of Environmental Science & Management University of California Santa Barbara California USA; ^3^ Joint BioEnergy Institute (JBEI) Emeryville California USA; ^4^ Department of Bioengineering University of California Santa Barbara California USA

**Keywords:** anaerobic communities, CAZymes, metagenome‐assembled genomes, plastics

## Abstract

Anaerobic microbial communities are often highly degradative, such as those found in the herbivore rumen and large‐scale anaerobic digesters. Since the microbial communities in these systems degrade recalcitrant organic polymers, we hypothesize that some microbes in anaerobic environments may be involved in man‐made plastic association, deformation, or even breakdown. While efforts have been put toward characterizing microbial communities, many microbes remain unidentified until they can be sufficiently cultivated to generate enough genetic material to assemble high‐quality metagenome assemblies and reference genomes. In this study, microbial consortia from goat fecal pellets and anaerobic digester sludge were cultivated for over 6 weeks to assemble metagenomes from novel anaerobic taxa with potential degradative activity. To select for microbes with potential plastic‐degrading abilities, plastic strips were included in culture, though the presence of plastic did not appear to enrich for particularly degradative consortia, yet it did select for novel species that otherwise may not have been characterized. Whole‐genome shotgun sequencing enabled assembly of 72 prokaryotic metagenome‐assembled genomes (MAGs) with >90% completion, <5% contamination, and an N50 >10,000 bp; 17 of these MAGs are classified as novel species given their lack of similarity to publicly available genomes and MAGs. These 72 MAGs vary in predicted carbohydrate‐degrading abilities, with genes predicted to encode fewer than 10 or up to nearly 400 carbohydrate‐active enzymes. Overall, this enrichment strategy enables characterization of less abundant MAGs in a community, and the MAGs identified here can be further mined to advance understanding of degradative anaerobic microbial consortia.

## 1 INTRODUCTION

Only a small percentage of microbes have been cultivated and characterized.^1^ Yet, microbes are involved in vast aspects of human life,^2^, ^3^ including the war on plastics.^4^ Microbes produce degradative enzymes, such as carbohydrate‐active enzymes (CAZymes) and rely on a carbon source for growth, which plastics can potentially offer.^5^ More than 5 billion tons of plastic waste can be found in the environment,^6^ and that number continues to grow. Current nonbiological methods to degrade these materials often demand high energy input—namely high pressures and temperatures.^7^ Conversely, numerous CAZymes are known to degrade organic plant‐based polymers under natural conditions throughout the environment.^8^, ^9^ A limited number of plastic‐degrading microbes have been identified, such as *Ideonella sakaiensis*,^10^ but numerous microbial communities found in degradative environments have not been adequately characterized, including those from anaerobic systems. Thus, a greater understanding of the microbial world, particularly of degradative microbial communities, will enhance society's ability to use biology to fight plastic pollution.

Degradative anaerobic microbial systems, including the guts of large herbivores and industrial anaerobic digesters, entail intricate interactions between community members. Still, individual members and metabolically diverse groups within the larger community must execute their roles for the community to function effectively. The microbes found in these communities are challenging to cultivate, but to characterize them (particularly less abundant strains), it is often necessary to cultivate them for sufficient DNA to assemble genomes.^11^ Both the guts of large herbivores and anaerobic digesters degrade recalcitrant substrates.^12^, ^13^ Anaerobes in the guts of large herbivores break down and metabolize plant biomass (lignocellulose), which contains three major polymers: cellulose, hemicellulose, and lignin. It has been suggested that observations from microbial lignocellulose breakdown can apply well to plastics breakdown.^14^ Lignin is structurally related to plastics, such as polystyrene and polyethylene terephthalate because these materials contain an abundance of carbon–carbon bonds and benzene rings.^15^ We hypothesize that lignin degraders might associate with, deform, or even degrade plastics because they are adapted to break down materials with similar chemical traits to plastics.

Additionally, a recent study showed the plastic‐degrading potential of the rumen by testing hydrolysis of plastics after exposure to rumen fluid.^16^ Certain ruminants also consume plastic on occasion unintentionally^17^ (or intentionally), and while this is a health concern,^18^ it invites further study into the specific plastic‐associating microbes and the other microbial members they interact with that might be found in ruminant guts. Industrial anaerobic digesters use microbial communities to degrade waste, such as lignocellulose, food waste, or materials found in wastewater.^19^ Plastics frequently contaminate these feed streams.^20^ Because of plastic exposure, it may be possible to find plastic‐associating and potentially plastic‐degrading microbes in anaerobic digesters.

Enzymes are a critical component involved in microbial degradation of plastics. If the microbe can both degrade and consume plastic, it is first necessary to secrete enzymes to break down the polymer into bite‐sized pieces which the cell can then take up.^21^ Enzymes of particular interest for plastic degradation include hydrocarbon‐degrading enzymes^22^ and certain CAZymes,^14^, ^23^ namely carbohydrate esterases.^24^ Anaerobic microbial consortia and their enzymatic machinery have not been adequately characterized, including communities found in the guts of large herbivores and industrial anaerobic digesters. Recent studies have drastically increased characterization of community members,^11^, ^25^ particularly efforts of the Hungate 1000 collection.^26^ However, further characterization would greatly benefit the community to overcome common challenges microbes contribute to in these environments, like instability in anaerobic digesters^27^ or gut problems in large herbivores.^28^

**FIGURE 1:**
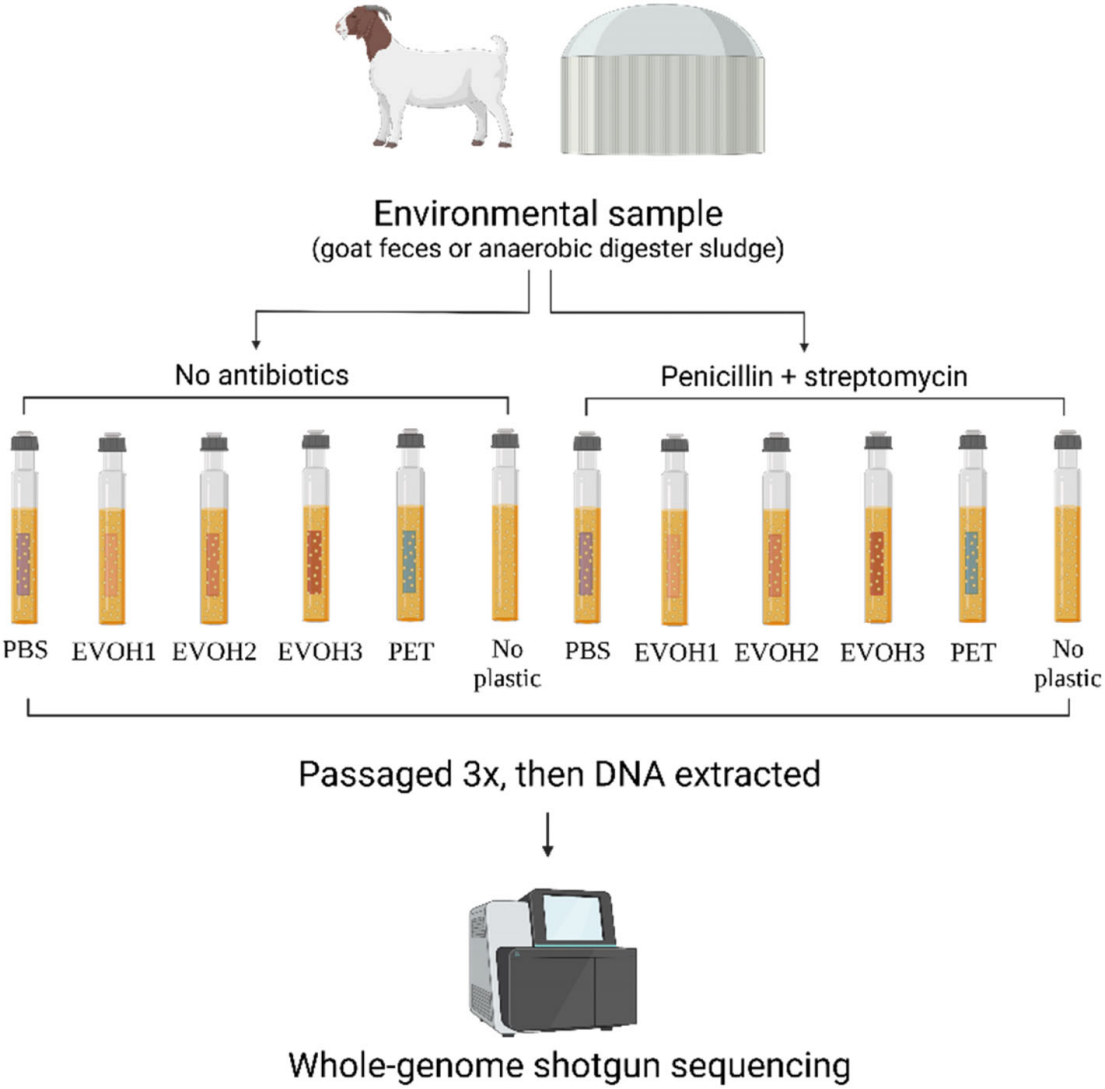
Experimental design for cultivation of anaerobic microbial communities and metagenome assembly of novel taxa. Environmental samples (goat fecal pellets or anaerobic digester sludge) were used to inoculate media containing a strip of specified plastic. Cultures were passaged 3× over about 6.5 weeks. DNA was then extracted from these communities and sequenced. EVOH1, ethylene vinyl alcohol copolymer (29 mol% ethylene); EVOH2, ethylene vinyl alcohol copolymer (38 mol% ethylene); EVOH3, ethylene vinyl alcohol copolymer (44 mol% ethylene); PBS, polybutylene succinate; PET, polyethylene terephthalate.

In this study, we sought to identify microbes found in anaerobic environments with genomes rich in degradative genes and previously uncharacterized species. We were particularly interested in identifying organisms that might carry high counts of genes encoding CAZymes, as these may enable microbes to degrade plastic. We cultivated microbial communities from goat fecal pellets and anaerobic digester sludge in culture media with a strip of synthetic plastic (ethylene vinyl alcohol copolymer, polyethylene terephthalate, or polybutylene succinate) for approximately 6 weeks (Figure 1). While no plastic degradation visibly occurred over the course of this experiment, communities grew sufficiently for DNA extraction, and that DNA was used for whole‐genome shotgun sequencing, which led to the assembly of 72 high‐quality metagenome‐assembled genomes (MAGs), several with hundreds of predicted CAZyme genes.

## 2 MATERIALS AND METHODS

### 2.1 Dilution and inoculation of source materials

Fresh goat fecal pellets were collected from the Orella Ranch (Goleta, CA, USA), and anaerobic digester sludge was obtained from the El Estero Water Resource Center (Santa Barbara, CA, USA) on November 30, 2020. Nine fecal pellets (5.1 g) were added to 30 mL MC– media^11^ in a 50 mL falcon tube and bubbled with CO_2_ to displace oxygen. MC– medium is modified from medium C,^29^ which was designed for anaerobic fungi, but MC– medium has been used to cultivate anaerobic fungi, methanogens, and anaerobic bacteria.^11^ Separately, 5 mL (5.1 g) anaerobic digester sludge was added to 20 mL MC– in a 50 mL falcon tube while bubbling with CO_2_. The falcon tubes were briefly bubbled with CO_2_, and then capped and vortexed to mix into a slurry. Approximately 1 mL of the slurry (either goat fecal pellet slurry or anaerobic digester sludge slurry) was added to individual 16 × 125 mm Hungate tubes containing 9 mL MC– media and 0.1 mL vitamin supplement (0.2 μm filtered; made in‐house following recipe from ATCC catalog no. MD‐VS and diluted 10× in Millipore water; final concentration of 0.1% v:v), except for controls with no inoculum. A sterile syringe‐needle anaerobic technique was used to inoculate the anaerobic digester sludge tubes, but the goat fecal pellet slurry was thicker, thus making it necessary to open the Hungate tube to add the inoculum instead of using a needle (which was done while bubbling with CO_2_). Hungate tubes were inverted to mix, and then pressures were zeroed using a pressure transducer. Hungate tubes were then incubated anaerobically at 39°C.

### 2.2 Plastic materials

Plastic materials were provided by Mitsubishi Chemical–Center for Advanced Materials (MC‐CAM). These included bio‐based polybutylene succinate (BioPBS™ (FZ Type)), polyethythene terephthalate (TAIKO Polyester Film), ethylene vinyl alcohol copolymer (SoarnoL DT2904RB, ET3803RB, and AT4403B), and linear low‐density polyethylene (LLDPE) (NOVATEC™‐LL UF230). Each small strip of plastic film (~ 4 cm × 0.7 cm) was disinfected with 70% ethanol prior to addition to Hungate tubes. Plastic strips were added to the Hungate tube containing culture media in an anaerobic chamber (catalog no. AS‐580, Anaerobe Systems, Morgan Hill, CA, USA), with headspace containing 5% hydrogen, 20% CO_2_, and balance nitrogen.

### 2.3 Enrichment and routine cultivation of anaerobic microbial communities

Microbes were cultivated in MC– media^11^ and incubated anaerobically at 39°C. After the initial enrichments grew for 11 days, they were passaged three more times, with each passage lasting 11–12 days. Passaging took place in an anaerobic chamber (catalog no. AS‐580, Anaerobe Systems). For the first two transfers, growing Hungate tube cultures were opened in the anaerobic chamber, and approximately 9 mL culture were removed using a sterile serological pipet. The plastic strip was left in the Hungate tube, along with approximately 1 mL of growing culture. Then, 9 mL fresh MC– media were added to the tube with a sterile serological pipet, and the Hungate tube was capped and then incubated anaerobically at 39°C. For the last transfer, the plastic strip was moved to a sterile 60 mL serum bottle containing 40 mL MC– media; the strip was moved using the end of a sterile serological pipet. In addition to the plastic strip, 1 mL inoculum from the 12‐day old growing Hungate tube was added to the serum bottle, and the serum bottle was then incubated at 39°C for 12 days.

### 2.4 Culture media additives

Vitamin supplement (made in‐house following recipe from ATCC catalog no. MD‐VS; final concentration of 0.1% v:v) was added to each culture vessel for each passage, including the initial enrichment from source materials. Penicillin–streptomycin (pen–strep, Thermo Fisher Scientific catalog no. 15–140‐122; penicillin final concentration of 100 units/mL and streptomycin final concentration of 100 μg/mL) was added to approximately half the samples (indicated by “PS” label) and was added each passage. Both vitamin supplement and penicillin–streptomycin were added at the start of each passage, except for the passage following the initial growth of microbial cultures from source materials. For this passage, penicillin–streptomycin was added the day after inoculation, and vitamin supplement was added 3 days after that.

### 2.5 LLDPE microbial enrichment

The microbial enrichment on LLDPE was slightly different than the other samples. Rather than inoculating a tube containing plastic directly from an environmental sample, this culture was inoculated with a microbial community previously grown from goat feces on medium C^30^ with reed canary grass as the substrate. It was then cryopreserved in 15% glycerol medium C and later revived and cultivated in the presence of LLDPE. It was then cryopreserved on LLDPE and revived and cultivated for DNA extractions. The initial dilution of source materials was similar to the other dilutions in this study. The LLDPE microbial consortium cryostock was revived in a serum bottle with 40 mL MC– and two strips of LLDPE (~4 cm × 0.7 cm), and this was used as a seed culture to inoculate two bottles of 40 mL MC– (one with two strips of LLDPE and one without any plastic). These cultures were grown for 6 days and then harvested the same way as the other cultures. Penicillin–streptomycin (at same concentration as described above) was added during early enrichment cultivation but not when revived and cultivated for DNA extraction.

### 2.6 DNA extractions

At the end of the third passage (inoculation referred to as passage 0), cultures were harvested by pouring the culture into a 50 mL falcon tube. The tubes were then centrifuged for 30 min at 4°C and 10,000 *g* using an Eppendorf rigid angle rotor centrifuge (F‐34‐6‐38). The supernatant was then removed, and the remaining cell pellet/plastic was stored at −80°C.

DNA was extracted using the FastDNA™ Spin Kit for Soil (MP Biomedicals) following manufacturer instruction, except the sodium phosphate buffer was added to the cell pellet to suspend the cells, and then that suspension was moved to the Lysing matrix E tube; homogenization was performed for 5 min at speed 10 using the Qiagen vortex adapter (catalog no. 13000‐V1‐24); the centrifugation step following homogenization was extended to 15 min; and after adding DES elution buffer (DNase/pyrogen‐free water), the tube was incubated at 55°C for 5 min prior to centrifugation. All centrifugation steps were done at 25°C in an Eppendorf rigid angle rotor centrifuge (F‐45‐30‐11). DNA was eluted in 100 μL DES. Quality control was performed using the Qubit 2.0 fluorometer and Agilent 2200 TapeStation, with typical concentrations ranging from tens to hundreds of nanograms per microliter.

### 2.7 High‐performance liquid chromatography

Immediately after transferring cultures to serum bottles for the last passage, 1 mL of the new culture was taken and stored at −20°C for initial time point high‐performance liquid chromatography (HPLC) measurements. At the end of this passage, 1 mL from each culture was removed and stored at −20°C for end point HPLC analysis. Samples were thawed and prepped for HPLC analysis by adding 50 μL of 50 mM H_2_SO_4_ to 500 μL sample. Acetate standards were made by adding sodium acetate to MC– media at concentrations of 0.1 g/L and 1.0 g/L, and 50 μL of 50 mM H_2_SO_4_ was added to 500 μL of each of these acetate standards. The samples were vortexed and then centrifuged at maximum speed at 25°C using an Eppendorf rigid angle rotor centrifuge (F‐45‐30‐11). Then, the supernatant was filtered through a 0.22 μm syringe filter (Millex) into a vial insert. These samples were then run on the HPLC (Agilent 1260 Infinity) with an autosampler unit (1260 ALS) and an organic acids column (Bio‐Rad Aminex HPX‐87H) with a 5 mM H_2_SO_4_ mobile phase as described previously,^11^ except the flowrate was 0.6 mL/min. The variable wavelength detector was employed for measurements. For acetate concentrations, the blank medium was subtracted off as it contained a small amount of acetate.

### 2.8 Library preparation and Illumina shotgun sequencing

Genomic DNA libraries were prepared for 24 samples for whole‐genome shotgun sequencing using the Nextera DNA prep kit and Nextera Index kit. Library quality control was performed using the Agilent 2200 TapeStation and Qubit 2.0 fluorometer. The library was sequenced on the Illumina NextSeq 500 mid output (paired‐end, 151 bp reads).

### 2.9 Metagenomic assembly and analysis

Reads were checked for quality control using FastQC v0.11.9.^31^ Reads from the four lanes per sample were then concatenated (forward reads separately from reverse reads) and then trimmed using Trimmomatic v.0.39^32^ with the following parameters for paired‐end trimming: ILLUMINACLIP:adapter.fasta:2:30:10:2:True LEADING:10 TRAILING:10 SLIDINGWINDOW:4:20 HEADCROP:20 CROP:148 MINLEN:36, where the adapter.fasta contained the Nextera paired‐end sequence: CTGTCTCTTATACACATCT. Only paired trimmed reads were used through the rest of the analysis. The reads were run on Kaiju v1.9.0^33^ using KBase default parameters^34^ for relative abundance of known taxa.

Prior to assembly, the SPAdes (v3.15.5) error correction tool^35^ was applied to the reads (without assembly). MEGAHIT (v.1.2.9)^36^ was then used to co‐assemble trimmed reads for each environment. MEGAHIT was run separately for enrichments from anaerobic digester sludge, enrichments from goat fecal pellets, and the enrichment from a previous cryostock. Anvi'o (v.7.1)^37^ was used to simplify the labels within the fasta coassemblies (anvi‐script‐reformat‐fasta with the parameter—simplify‐names).

Trimmed reads from samples associated with each co‐assembly were aligned to the corresponding MEGAHIT assembly using Bowtie2 v2.4.5^38^ with the parameter—sensitive; for the enrichment from a previous cryostock, the same parameters were used, but the reads used for the alignment were both trimmed and error corrected. The output .sam file was sorted and converted to a .bam file using SAMtools v1.14.^39^ Binning was performed using MetaBat 2 v2.15^40^ with default parameters. Bins from all environments were then combined and dereplicated using dRep v3.4.0 dereplicate^41^ with CheckM^42^ with the following parameters: ‐comp 90 ‐con 10 ‐sa 0.95. Only one MAG had contamination between 5% and 10%, and it was removed to increase MAG standards to include only MAGs with <5% contamination. GTDB‐Tk v2.3.0 classify_wf^43^ was used to classify MAGs and identify which were novel at the species level, using the Genome Taxonomy Database vR214 as the reference database.^44^ The binned MAGs were transferred to KBase^34^ and checked on QUAST v4.4^45^ and removed if the N50 was lower than 10,000 bp. MAGs were annotated using RASTtk v1.073^46^ with default KBase parameters except that archaeal bins were set to the domain: archaea. On KBase, MAGs and reference genomes/MAGs were searched for predicted CAZyme genes using dbCAN2 v10.^47^ Substrate predictions for CAZymes were made using the dbCAN server (accessed 12/21/23–12/22/23) with the HMMER: dbCAN‐sub output.^48^ CoverM v0.6.1 (https://github.com/wwood/CoverM) was used in the genome mode with the relative abundance method (with minimum covered fraction set to 0) to calculate the relative abundance of sample reads mapped to each of the MAGs.

To check for eukaryotes, raw, concatenated, paired‐end reads were run on EukDetect^49^ (installed March 20, 2024 with the database: “eukdetect_database_metarch_v2” version 9), with the mode “runall.”

### 2.10 Phylogenetic tree

To make the phylogenetic tree, we used the FastANI reference provided by GTDB‐Tk (v.2.3.0)^43^ for each MAG or the closest placement reference if no FastANI reference was available. Certain novel MAGs were not associated with either a FastANI reference or a closest placement reference, and thus no reference was added to the tree. Reference genomes and MAGs were imported from RefSeq^50^ and Genbank.^51^ MAGs and their associated references were run on the KBase tool: Insert Genome Into SpeciesTree v.2.2.0,^34^, ^52^ set to find one nearest neighbor; this nearest neighbor was removed in the visualization step as it was a redundant reference. This tree was visualized using iTOL v6.^53^ MAG labels were decided based on the GTDB‐Tk classification but using the corresponding NCBI name. As the novel MAGs did not have a corresponding NCBI classification, the tree names were determined by finding the most specific GTDB‐Tk taxonomic classification that is listed as a taxonomic classification on the NCBI website. The MAGs were designated with “ADS” followed by a number between 1 and 36 for the 36 MAGs assembled from anaerobic digester sludge in this study. The 35 MAGs assembled from the ruminant (goat) fecal sample enrichments in this study are designated with “RFS” and a number between 1 and 35. The one MAG assembled from the LLDPE enrichment from the goat fecal enrichment cryostock revival is designated as “RFSC1.”

### 2.11 Assessing protein homology to known plastic‐degrading enzymes

We used the Protein BLAST server^54^ on May 15, 2024 with default parameters to align annotated α/β hydrolases from the MAGs to several known plastic‐degrading enzymes found in the PAZy database.^24^ Specifically, we compared against the following enzymes (listed with their NCBI designation): prolyl oligopeptidase family serine peptidase from *Bacillaceae* (WP_041846030.1),^55^ putative esterase YtxM from *Caldibacillus thermoamylovorans* (CEE00769.1),^55^ α/β hydrolase from Candidatus Bathyarchaeota archaeon MAG (RLI42440.1),^56^ and α/β fold hydrolases from *Holdemanella biformis* (WP_118011433.1),^57^ *Alcanivorax borkumensis* (Q0VLQ1),^58^ *Bacillaceae* (WP_034767800.1),^55^ and Clostridiales bacterium AM23‐16LB (RGD93181.1).^57^ We filtered results to include only those with at least 25% identity, an *E*‐value less than 10^−5^, and query coverage between 50% and 100%. We kept only results where these restrictions were met whether the reference sequence or MAG translated protein from this study were used as the query; MAG translated proteins were used as the query for results listed in Supplemental File 1. We also ran translated protein sequences of each annotated α/β hydrolase from the MAGs and reference protein sequences on InterPro^59^ with default parameters on May 13, 2024 in search of Pfam domains^60^ for the sequences.

## 3 RESULTS AND DISCUSSION

We used the Illumina NextSeq 500 to sequence 24 samples: 12 with inoculum from anaerobic digester sludge and 12 with inoculum from goat fecal pellets. Approximately half of the cultures were treated with antibiotics (penicillin + streptomycin) to select for microbes that might be overgrown by abundant bacteria; the other half were not treated with antibiotics to allow any strains to grow that could. Each culture contained one strip of plastic (ethylene vinyl alcohol copolymer, polyethylene terephthalate, polybutylene succinate, or LLDPE) except for controls that were inoculated but contained no plastic (Supplemental Table 1). From the whole‐genome shotgun sequencing data, we co‐assembled MAGs from each environment. We used MEGAHIT^36^ for assembly and then binned MAGs using MetaBAT 2.^40^ After dereplication and quality control using dRep^41^ and CheckM,^42^ our dataset included dozens of MAGs with high completion and low contamination. Using GTDB‐Tk,^43^ we identified several of these MAGs as novel species based on lack of sequence similarity to known species.

### 3.1 MAGs contribute to prokaryotic phylogenetic tree

Although some argument exists for the species sequence similarity cutoff,^61^ we dereplicated our MAGs at 95% average nucleotide identity. Our co‐assembly and dereplication process yielded 73 MAGs with >90% completion and <5% contamination. One MAG was then excluded because it had an N50 <10,000 bp. MAG sizes range from just over 1 Mbp to over 5 Mbp. Some MAGs are GC‐rich, whereas others are AT‐rich, but the average is close to 50% GC (Supplemental Table 2). Many of these MAGs align with anaerobes previously found in anaerobic digester and ruminant environments.^11^, ^25^, ^62^ Bacterial MAGs are associated with both gram‐positive and gram‐negative species, and numerous MAGs are classified as Clostridia or Bacteroidia at the class level.

**FIGURE 2:**
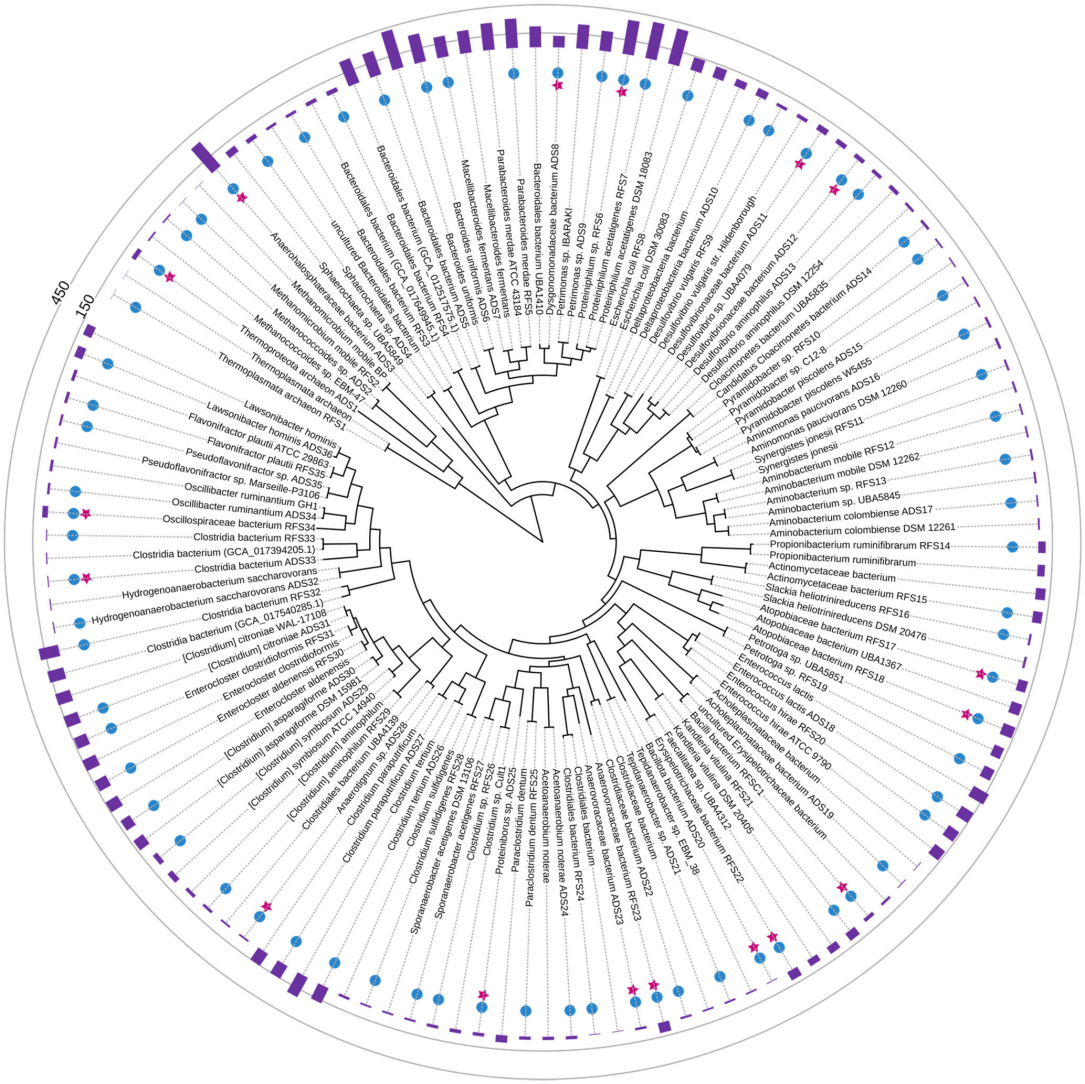
Species tree containing 72 metagenome‐assembled genomes (MAGs) from this study. Produced using SpeciesTree^52^ app in KBase^34^ with references identified from GTDB‐Tk^43^ and visualized with iTOL.^53^ Several MAGs cluster with known anaerobes, including many common to the ruminant environment. Purple bars are the number of CAZymes predicted by dbCAN2^47^ via KBase;^34^ blue dots represent MAGs assembled in this study; pink stars represent novel MAGs identified in this study. Interactive tree can be visualized online at: https://itol.embl.de/tree/7019188200488621691816597. Accession numbers for reference genomes and MAGs are listed in Supplemental Table 5.

These MAGs add to the prokaryotic tree of life, with some aligning closely to well‐characterized anaerobes, whereas others are newly characterized (Figure 2). Seventeen of these MAGs are classified as novel species according to GTDB‐Tk v.2.3.0;^43^ sixteen of these novel MAGs are bacterial, while one is archaeal—detailed taxonomic classifications are shown in Table 1. Based on the EukDetect pipeline,^49^ eukaryotes were not detected in the sequencing reads.

**TABLE 1:** Basic information for novel MAGs assembled in this study.

MAG label	Classification (GTDB‐Tk)	MAG size (Mbp)^a^	# Coding genes^a^	% GC^b^
Thermoproteota archaeon ADS1	Domain: Archaea; phylum: Thermoproteota; class: Bathyarchaeia; order: B26‐1; family: UBA233; genus: PALSA‐986; species__	2.74	3129	42.6
Anaerohalosphaeraceae bacterium ADS3	Domain: Bacteria; phylum: Planctomycetota; class: Phycisphaerae; order: Sedimentisphaerales; family: Anaerohalosphaeraceae; genus: UBA7708; species__	3.11	2742	51.6
Dysgonomonadaceae bacterium ADS8	Domain: Bacteria; phylum: Bacteroidota; class: Bacteroidia; order: Bacteroidales; family: Dysgonomonadaceae; genus: UBA4179; species__	1.75	1751	41.0
Desulfovibrionaceae bacterium ADS11	Domain: Bacteria; phylum: Desulfobacterota; class: Desulfovibrionia; order: Desulfovibrionales; family: Desulfovibrionaceae; genus__; species__	3.78	3694	64.7
Desulfovibrionaceae bacterium ADS12	Domain: Bacteria; phylum: Desulfobacterota; class: Desulfovibrionia; order: Desulfovibrionales; f__Desulfovibrionaceae; genus: Aminidesulfovibrio; species__	3.08	3128	68.5
Bacillota bacterium ADS20	Domain: Bacteria; phylum: Bacillota_B; class: Peptococcia; order: DRI‐13; family: UBA5745; genus__; species__	2.98	3046	54.3
Anaerovoracaceae bacterium ADS23	Domain: Bacteria; phylum: Bacillota_A; class: Clostridia; order: Peptostreptococcales; family: Anaerovoracaceae; genus__; species__	2.44	2457	35.4
Proteiniborus sp. ADS25	Domain: Bacteria; phylum: Bacillota_A; class: Clostridia; order: Tissierellales; family: Proteiniboraceae; genus: Proteiniborus; species__	3.28	3286	31.9
Anaerotignum sp. ADS28	Domain: Bacteria; phylum: Bacillota_A; class: Clostridia; order: Lachnospirales; family: Anaerotignaceae; genus: Anaerotignum; species__	2.55	2561	39.0
Clostridia bacterium ADS33	Domain: Bacteria; phylum: Bacillota_A; class: Clostridia; order: Oscillospirales; family: Butyricicoccaceae; genus__; species__	2.54	2704	51.9
Proteiniphilum sp. RFS6	Domain: Bacteria; phylum: Bacteroidota; class: Bacteroidia; order: Bacteroidales; family: Dysgonomonadaceae; genus: Proteiniphilum; species__	4.23	3658	44.2
Atopobiaceae bacterium RFS17	Domain: Bacteria; phylum: Actinomycetota; class: Coriobacteriia; order: Coriobacteriales; family: Atopobiaceae; genus: RUG721; species__	2.04	1943	63.6
Atopobiaceae bacterium RFS18	Domain: Bacteria; phylum: Actinomycetota; class: Coriobacteriia; order: Coriobacteriales; family: Atopobiaceae; genus: UBA1367; species__	2.46	2204	65.2
Erysipelotrichaceae bacterium RFS22	Domain: Bacteria; phylum: Bacillota; class: Bacilli; order: Erysipelotrichales; family: Erysipelotrichaceae; genus: UBA4312; species__	2.35	2431	42.8
Anaerovoracaceae bacterium RFS23	Domain: Bacteria; phylum: Bacillota_A; class: Clostridia; order: Peptostreptococcales; family: Anaerovoracaceae; genus: RUG099; species__	2.59	2444	48.9
Oscillospiraceae bacterium RFS34	Domain: Bacteria; phylum: Bacillota_A; class: Clostridia; order: Oscillospirales; family: Oscillospiraceae; genus: ER4; species__	2.25	2228	60.5
Bacilli bacterium RFSC1	Domain: Bacteria; phylum: Bacillota; class: Bacilli; order: RFN20; family: CAG‐826; genus: RUG14515; species__	1.38	1224	35.9

^a^ Determined from RASTtk v.1.073^46^ via KBase.^34^

^b^ Determined from QUAST v.4.4^45^ via KBase.^34^

### 3.2 MAGs are predicted to have varying polymer‐degrading capabilities

Several MAGs are predicted to encode hundreds of CAZymes, whereas others contain fewer than 10 predicted CAZyme genes (Figure 2). The MAGs with highest predicted CAZyme counts mainly encode glycoside hydrolases. This makes sense given the environments where samples were taken from and used for inoculation sources. For instance, cellulose must be degraded in the guts of large herbivores, making cellulases crucial. Genes predicted to encode carbohydrate esterases and polysaccharide lyases were also found among numerous MAGs (Supplemental Table 3). Additionally, two MAGs were predicted to encode for an enzyme in the auxiliary activity family 1, which includes laccases that have been associated with plastic‐degrading potential.^63^, ^64^

**FIGURE 3:**
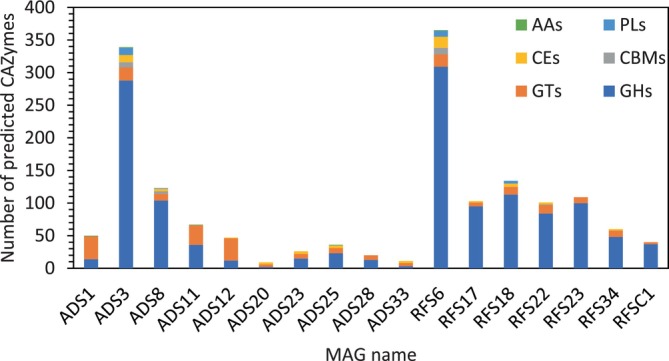
Novel metagenome‐assembled genomes (MAGs) have varying predicted CAZyme profiles, but largest quantities belong to glycoside hydrolase and glycosyltransferase families. MAG names contain MAG designation; full names are shown in Table 1. AAs, auxiliary activities; CBMs, carbohydrate binding modules; CEs, carbohydrate esterases; GHs, glycoside hydrolases; GTs, glycosyl transferases; PLs, polysaccharide lyases.

Two novel MAGs, Anaerohalosphaeraceae bacterium ADS3 and *Proteiniphilum* sp. RFS6, encode for >300 predicted CAZymes each (Figure 3). According to dbCAN‐sub,^48^ the diverse set of CAZymes within each of these two MAGs are predicted to act on more than 20 different substrates, though the majority annotated are associated with xylan, host glycan, and beta‐glucan (Supplementary File 1). Among the 72 MAGs assembled in this study, *Proteiniphilum* sp. RFS6 encodes the greatest numbers of predicted carbohydrate binding modules and carbohydrate esterases (Supplemental Table 3). The high carbohydrate esterase count is particularly interesting because this family contains enzymes belonging to the Enzyme Class 3.1 (E.C. 3.1.‐.‐),^65^, ^66^ which contains α/β hydrolases, and is where current plastic‐degrading enzymes often fit.^24^

Close to half the MAGs found in this study contain one or more genes annotated as an α/β hydrolase. In an effort to identify homologs to known plastic‐degrading enzymes, we used BlastP^54^ to align these α/β hydrolases to enzymes found in the plastics‐active enzymes (PAZy) database^24^ and InterPro^59^ to evaluate Pfam domains.^60^ Several MAGs from this study, including novel MAGs, encode an α/β hydrolase with significant sequence similarity to one or more known plastics‐active enzymes (Supplemental File 1). Highest similarity scores were for three translated MAG proteins compared to the α/β fold hydrolase from *H. biformis* (NCBI reference: WP_118011433.1), a polyethylene terephthalate‐active enzyme.^57^ The top three hits had >50% identity and almost complete coverage (>90%). The same three translated MAG proteins had >30% similarity and high coverage compared to an archaeal MAG α/β hydrolase (NCBI reference: RLI42440.1) that is also active against polyethylene terephthalate.^56^ The top hit had the same Pfam domain (serine aminopeptidase, S33 (PF12146)) as the *H. biformis* sequence, whereas the other two highest hits shared their Pfam domain (prolyl oligopeptidase family (PF00326)) with the archaeal reference protein sequence. These different proteins are presumably homologous, and the three top hit MAG translated proteins mentioned from this study may also be active against polyethylene terephthalate. In addition, numerous other translated MAG proteins have >25% identity, >70% coverage, *E* values below 10^−7^, and the same Pfam domain as a putative esterase from *C. thermoamylovorans* (NCBI reference: CEE00769.1) and/or an α/β hydrolase from *A. borkumensis* (NCBI reference: Q0VLQ1), which have been shown to be active on polyethylene terephthalate and polylactic acid, respectively.^55^, ^58^ While percent identity may seem low in some of these cases, that is not uncommon among homologous proteins.^67^ Given the low *E* values, high coverage, and reasonable percent identities for several proteins encoded in the MAGs from this study compared to reference plastics‐active enzymes, especially when the sequences share their Pfam domain with the reference, it is likely these proteins are homologous to plastics‐active enzymes.

Although several MAGs show high degradation potential, the five most abundant MAGs (based on median relative abundance among the samples) have genes predicted for less than 50 CAZymes each (Supplemental Table 3 and Supplemental File 1). Many of the microbes with low CAZyme gene counts can likely be termed “scrappy microbes” as they grew in media without a sugar source and presumably survived on media components. There were also likely microbes commonly known as “cheaters” that utilized degradation products from their fellow microbial members.^68^ Metabolic exchange is key within these communities,^69^ hence the ability of certain members to survive without much degradative machinery. The culture conditions and inoculation sources also likely played a major role in the variability of degradation capacities.

### 3.3 Plastic enrichments select for cultivable microbes

The most abundant MAG had a median relative abundance around 8% and mean around 10% (Supplemental File 1). It was classified as the same species as *Aminobacterium* sp. UBA5845.^70^ This species was previously sequenced directly from an environmental sample without in vitro growth;^70^ however, this species was also obtained from a cultivation study that used environmental samples from wastewater sludge.^71^ The second most abundant MAG was classified as *Desulfovibrio vulgaris*, a common model microbe.^72^ Model microbes are meant to be easy to cultivate and work with; it makes sense that a cultivation study would show the presence of such microbes.

Cultivation experiments like this one are useful to identify microbes that can be grown in vitro and thus further studied. In addition to sequencing DNA, pressure measurements and acetate production provide further evidence for the growth of these microbes in vitro (Supplemental Figures 1 and 2). For future studies, the plastic strips could be removed given that the microbes likely did not rely on them for growth, and they present a potential source of contamination when only disinfected with ethanol. Proper sterilization of nonautoclavable plastics is often cost‐prohibitive in lab‐scale studies, making ethanol disinfection a common but insufficient attempt at sterilization,^73^, ^74^ as was seen in this study where four controls provided sufficient DNA for sequencing. However, this study focused on providing MAGs, and thus microbial contamination of the plastics was not prohibitive. For plastic association and degradation experiments, plastic strips are relevant to better understand microbes with potential plastic association, but culture media should be as minimal as possible, as this will limit the total number of microbes that can grow and will be more selective in the enrichment.

Including plastics in this enrichment study enabled ease of assembly of several microbial genomes that otherwise may not have been recovered. For example, *Pseudoflavonifractor* sp. ADS35 and *Lawsonibacter hominis* ADS36 were enriched on multiple EVOH samples from the anaerobic digester sludge inoculum (Supplemental File 1). Similarly, *Enterocloster aldenensis* RFS30 was abundant in several rumen‐derived consortia cultivated with plastics and antibiotics, but its relative abundance within the corresponding inoculated control without plastic (NS‐R‐PS) was orders of magnitude lower. Several novel MAGs were also enriched in consortia grown with plastic. For instance, Anaerovoracaceae bacterium RFS23 had a relative abundance of about 3% in the penicillin–streptomycin treated community grown from goat feces with EVOH3 (EVOH3‐R‐PS), while its relative abundance in the similarly treated sample without plastic (NS‐R‐PS) was only around 0.04%. Likewise, *Proteiniborus* sp. ADS25 had its highest relative abundance within an anerobic digester‐derived community with plastic present (EVOH2‐AD‐NA), and its relative abundance was about three orders of magnitude lower in the anaerobic digester cultures without plastic (NS‐AD‐PS and NS‐AD‐NA; Supplemental File 1). Microbial members may have been enriched due to their association with plastics, while others may have tagged along and grown on metabolic products. Still, plastics played a clear role in enriching certain members of the starting community.

**FIGURE 4:**
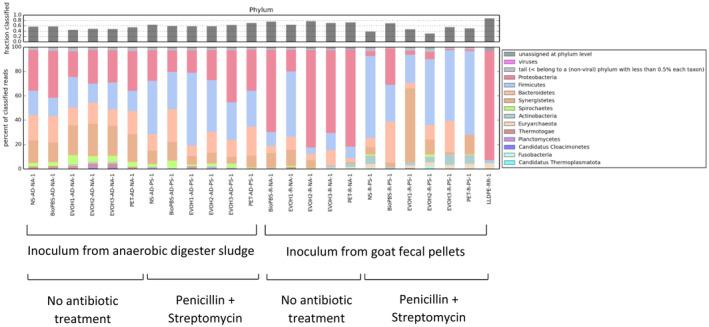
Microbial species abundances under the indicated enrichment conditions estimated using Kaiju^33^ on KBase.^34^ Reads from the 24 samples were trimmed and provided to Kaiju, and abundances at the phylum level show an abundance of Proteobacteria, Firmicutes, Bacteroidetes, and Synergistetes. Sample labels are defined in Supplemental Table 1.

Despite enrichment of certain MAGs with the presence of plastics, the type of plastic did not appear to have a major impact on the overall microbial communities enriched (Figure 4). The purpose of this experiment was more explorative and aimed at cultivating and characterizing novel taxa through metagenome assemblies following growth, but biological replicates on each type of plastic will be useful in future work. Trends in microbial communities from the sample reads appeared to depend mostly on inoculation source and antibiotics (Figure 4). This is also supported when mapping the sample reads to the MAGs. For example, when reads are mapped to three of the top five predicted CAZyme producing MAGs, reads from multiple samples from one inoculation source—either goat fecal pellets or anaerobic digester sludge—are found at >1% relative abundance, and the reads of all samples from the other environment have <1% relative abundance mapped to that MAG. Reads mapped to one such MAG are found at >1% relative abundance in every anaerobic digester sludge enrichment treated with penicillin and streptomycin but none of the other samples (Supplemental Table 3 and Supplemental File 1). These clustering patterns make sense given how microbial communities vary across different environments and are affected by antibiotic treatment.

## 4 CONCLUSIONS

Though the microbes identified in this study are only a small portion of the community present in the environments they were sampled from, the 72 high‐quality MAGs (and particularly the 17 novel MAGs) contribute to better understanding these environments and are present for public use. It is feasible that cultivation played a key role in assembling novel MAGs because these microbes were likely enriched during cultivation and thus were abundant enough in the community to obtain adequate sequencing coverage. Microbes in these communities vary from highly degradative to rather “scrappy” as was seen in the CAZyme gene profiles for the MAGs. Some of these scrappy microbes would likely also be considered “cheaters” as they may have survived off the products of other microbes rather than only the media components. Although this study captured a specific set of culture conditions, future work can include using different enrichment procedures to identify microbes from these environments that were not selected for or were outcompeted because of the culture media, temperature, pH, or other variables. Ultimately, through this and other studies, anaerobic degradative consortia can be better characterized and then used to combat challenges such as plastic pollution.

## AUTHOR CONTRIBUTIONS

**Elaina M. Blair:** Conceptualization; investigation; writing – original draft; methodology; visualization; validation; writing – review and editing; formal analysis; data curation. **Jennifer L. Brown:** Investigation; methodology. **Dong Li:** Writing – review and editing; validation; methodology; investigation; formal analysis; data curation. **Patricia A. Holden:** Writing – review and editing; investigation; funding acquisition; supervision; methodology; project administration. **Michelle A. O'Malley:** Methodology; writing – review and editing; funding acquisition; supervision; project administration; formal analysis.

## CONFLICT OF INTEREST STATEMENT

The authors declare no conflict of interest.

## Supporting information


**Data S1.** Supporting information.

**Data S2.** Supporting information.

## Data Availability

Raw reads are deposited in the NIH Sequence Read Archive under the BioProject: PRJNA1023249. Metagenome‐assembled genomes are also deposited under the same BioProject. MAG annotations from KBase are available at: https://kbase.us/n/176333/6/.
