# Supplementary material for: Metagenomics analysis yields assembled genomes from prokaryotic anaerobes with polymer‐degrading potential
Source: Biotechnol Prog. 2024 Jun 17;40(6):e3484. doi: 10.1002/btpr.3484 (PMC11659807; doi:10.1002/btpr.3484)
Supplement: Supplementary file 2 — Data S2. Supporting information. [file BTPR-40-e3484-s002.docx]

Supplementary Information

**Supplemental File 1.** Sheet 1: Relative abundances of trimmed sample reads mapped to each MAG using CoverM v0.6.1 (<https://github.com/wwood/CoverM>). Sheet 2: BlastP^1^ results and Pfam domains^2^ for MAG annotated α/β-hydrolases aligned to known plastic-degrading enzymes. Sheets 3-4: Output from dbCAN-sub^3^ for *Anaerohalosphaeraceae* bacterium ADS3 and *Proteiniphilum* sp. RFS6.

**Table 1.** Description of microbial enrichments from environmental samples.

| Sample Name | Plastic Type | Inoculum Source | Antibiotic Treatment |
| --- | --- | --- | --- |
| BioPBS-AD-NA | Polybutylene succinate | Anaerobic digester sludge | No antibiotics added |
| EVOH3-AD-NA | Ethylene vinyl alcohol copolymer (44 mole % ethylene) | Anaerobic digester sludge | No antibiotics added |
| EVOH1-AD-PS | Ethylene vinyl alcohol copolymer (29 mole % ethylene) | Anaerobic digester sludge | Penicillin + streptomycin |
| BioPBS-R-NA | Polybutylene succinate | Goat feces | No antibiotics added |
| EVOH3-R-NA | Ethylene vinyl alcohol copolymer (44 mole % ethylene) | Goat feces | No antibiotics added |
| EVOH2-R-PS | Ethylene vinyl alcohol copolymer (38 mole % ethylene) | Goat feces | Penicillin + streptomycin |
| PET-AD-NA | Polyethylene terephthalate | Anaerobic digester sludge | No antibiotics added |
| NS-AD-NA | No plastic | Anaerobic digester sludge | No antibiotics added |
| EVOH2-AD-PS | Ethylene vinyl alcohol copolymer (38 mole % ethylene) | Anaerobic digester sludge | Penicillin + streptomycin |
| PET-R-NA | Polyethylene terephthalate | Goat feces | No antibiotics added |
| BioPBS-R-PS | Polybutylene succinate | Goat feces | Penicillin + streptomycin |
| EVOH3-R-PS | Ethylene vinyl alcohol copolymer (44 mole % ethylene) | Goat feces | Penicillin + streptomycin |
| EVOH1-AD-NA | Ethylene vinyl alcohol copolymer (29 mole % ethylene) | Anaerobic digester sludge | No antibiotics added |
| BioPBS-AD-PS | Polybutylene succinate | Anaerobic digester sludge | Penicillin + streptomycin |
| EVOH3-AD-PS | Ethylene vinyl alcohol copolymer (44 mole % ethylene) | Anaerobic digester sludge | Penicillin + streptomycin |
| EVOH1-R-NA | Ethylene vinyl alcohol copolymer (29 mole % ethylene) | Goat feces | No antibiotics |
| PET-R-PS | Polyethylene terephthalate | Goat feces | Penicillin + streptomycin |
| NS-R-PS | No plastic | Goat feces | Penicillin + streptomycin |
| EVOH2-AD-NA | Ethylene vinyl alcohol copolymer (38 mole % ethylene) | Anaerobic digester sludge | No antibiotics added |
| PET-AD-PS | Polyethylene terephthalate | Anaerobic digester sludge | Penicillin + streptomycin |
| NS-AD-PS | No plastic | Anaerobic digester sludge | Penicillin + streptomycin |
| EVOH2-R-NA | Ethylene vinyl alcohol copolymer (38 mole % ethylene) | Goat feces | No antibiotics added |
| EVOH1-R-PS | Ethylene vinyl alcohol copolymer (29 mole % ethylene) | Goat feces | Penicillin + streptomycin |
| LLDPE-RR | Linear low-density polyethylene | Goat feces enrichment cryostock | Initially penicillin + streptomycin but then no antibiotics |

**Table 2**. Basic information for MAGs assembled in this study that have been previously characterized at the species level.

| MAG label | Classification (GTDB-Tk) | MAG size (Mbp)^a^ | Number of coding genes^a^ | %GC^b^ |
| --- | --- | --- | --- | --- |
| Methanococcoides sp. ADS2 | Domain: Archaea; phylum: Halobacteriota; class: Methanosarcinia; order: Methanosarcinales; family: Methanosarcinaceae; genus: Methanomethylovorans; species: Methanomethylovorans sp001896725 | 1.79 | 1966 | 42.5 |
| Sphaerochaeta sp. ADS4 | Domain: Bacteria; phylum: Spirochaetota; class: Spirochaetia; order: Sphaerochaetales; family: Sphaerochaetaceae; genus: Sphaerochaeta; species: Sphaerochaeta sp002432715 | 2.26 | 2237 | 51.8 |
| Bacteroidales bacterium ADS5 | Domain: Bacteria; phylum: Bacteroidota; class: Bacteroidia; order: Bacteroidales; family: Paludibacteraceae; genus: MVYY01; species: MVYY01 sp012517575 | 2.35 | 2264 | 37.9 |
| Bacteroides uniformis ADS6 | Domain: Bacteria; phylum: Bacteroidota; class: Bacteroidia; order: Bacteroidales; family: Bacteroidaceae; genus: Bacteroides; species: Bacteroides uniformis | 3.38 | 3036 | 47.1 |
| Macellibacteroides fermentans ADS7 | Domain: Bacteria; phylum: Bacteroidota; class: Bacteroidia; order: Bacteroidales; family: Tannerellaceae; genus: Macellibacteroides; species: Macellibacteroides fermentans | 3.07 | 2785 | 42.4 |
| Petrimonas sp. ADS9 | Domain: Bacteria; phylum: Bacteroidota; class: Bacteroidia; order: Bacteroidales; family: Dysgonomonadaceae; genus: Petrimonas; species: Petrimonas sp002356435 | 3.18 | 2898 | 44.5 |
| Deltaproteobacteria bacterium ADS10 | Domain: Bacteria; phylum: Desulfobacterota; class: Syntrophobacteria; order: Syntrophobacterales; family: Syntrophobacteraceae; genus: Desulforhabdus; species: Desulforhabdus sp012517535 | 4.19 | 4453 | 52.1 |
| Desulfovibrio aminophilus ADS13 | Domain: Bacteria; phylum: Desulfobacterota; class: Desulfovibrionia; order: Desulfovibrionales; family: Desulfovibrionaceae; genus: Aminidesulfovibrio; species: Aminidesulfovibrio aminophilus | 3.17 | 3246 | 66.6 |
| Candidatus Cloacimonetes bacterium ADS14 | Domain: Bacteria; phylum: Cloacimonadota; class: Cloacimonadia; order: Cloacimonadales; family: Cloacimonadaceae; genus: Cloacimonas; species: Cloacimonas sp002432865 | 2.20 | 1846 | 36.7 |
| Pyramidobacter piscolens ADS15 | Domain: Bacteria; phylum: Synergistota; class: Synergistia; order: Synergistales; family: Dethiosulfovibrionaceae; genus: Pyramidobacter; species: Pyramidobacter piscolens | 2.46 | 2547 | 60.0 |
| Aminomonas paucivorans ADS16 | Domain: Bacteria; phylum: Synergistota; class: Synergistia; order: Synergistales; family: Synergistaceae; genus: Aminomonas; species: Aminomonas paucivorans | 2.39 | 2452 | 68.1 |
| Aminobacterium colombiense ADS17 | Domain: Bacteria; phylum: Synergistota; class: Synergistia; order: Synergistales; family: Aminobacteriaceae; genus: Aminobacterium; species: Aminobacterium colombiense | 1.82 | 1861 | 45.1 |
| Enterococcus lactis ADS18 | Domain: Bacteria; phylum: Bacillota; class: Bacilli; order: Lactobacillales; family: Enterococcaceae; genus: Enterococcus_B; species: Enterococcus_B lactis | 2.75 | 2896 | 38.2 |
| Acholeplasmataceae bacterium ADS19 | Domain: Bacteria; phylum: Bacillota; class: Bacilli; order: ML615J-28; family: CAG-698; genus: DUNF01; species: DUNF01 sp012839485 | 1.16 | 1092 | 30.2 |
| Tepidanaerobacter sp. ADS21 | Domain: Bacteria; phylum: Bacillota_A; class: Thermosediminibacteria; order: Thermosediminibacterales; family: Tepidanaerobacteraceae; genus: Tepidanaerobacter; species: Tepidanaerobacter sp001896545 | 2.14 | 2258 | 37.4 |
| Clostridiaceae bacterium ADS22 | Domain: Bacteria; phylum: Bacillota_A; class: Clostridia; order: Lutisporales; family: Lutisporaceae; genus: UBA7994; species: UBA7994 sp003527145 | 4.13 | 4110 | 39.6 |
| Acetoanaerobium noterae ADS24 | Domain: Bacteria; phylum: Bacillota_A; class: Clostridia; order: Peptostreptococcales; family: Filifactoraceae; genus: Acetoanaerobium; species: Acetoanaerobium noterae | 2.80 | 2687 | 33.2 |
| Clostridium tertium ADS26 | Domain: Bacteria; phylum: Bacillota_A; class: Clostridia; order: Clostridiales; family: Clostridiaceae; genus: Clostridium; species: Clostridium tertium | 3.27 | 3216 | 27.8 |
| Clostridium paraputrificum ADS27 | Domain: Bacteria; phylum: Bacillota_A; class: Clostridia; order: Clostridiales; family: Clostridiaceae; genus: Clostridium; species: Clostridium paraputrificum | 3.33 | 3253 | 29.9 |
| [Clostridium] symbiosum ADS29 | Domain: Bacteria; phylum: Bacillota_A; class: Clostridia; order: Lachnospirales; family: Lachnospiraceae; genus: Clostridium_Q; species: Clostridium_Q symbiosum | 4.31 | 4279 | 48.1 |
| [Clostridium] asparagiforme ADS30 | Domain: Bacteria; phylum: Bacillota_A; class: Clostridia; order: Lachnospirales; family: Lachnospiraceae; genus: Enterocloster; species: Enterocloster asparagiformis | 5.34 | 5322 | 56.3 |
| [Clostridium] citroniae ADS31 | Domain: Bacteria; phylum: Bacillota_A; class: Clostridia; order: Lachnospirales; family: Lachnospiraceae; genus: Enterocloster; species: Enterocloster citroniae | 5.64 | 5372 | 49.8 |
| Hydrogenoanaerobacterium saccharovorans ADS32 | Domain: Bacteria; phylum: Bacillota_A; class: Clostridia; order: Oscillospirales; family: Ruminococcaceae; genus: Avimicrobium; species: Avimicrobium caecorum | 2.15 | 2336 | 56.9 |
| Oscillibacter ruminantium ADS34 | Domain: Bacteria; phylum: Bacillota_A; class: Clostridia; order: Oscillospirales; family: Oscillospiraceae; genus: Oscillibacter; species: Oscillibacter ruminantium | 3.03 | 3254 | 55.2 |
| Pseudoflavonifractor sp. ADS35 | Domain: Bacteria; phylum: Bacillota_A; class: Clostridia; order: Oscillospirales; family: Oscillospiraceae; genus: Marseille-P3106; species: Marseille-P3106 sp900169975 | 2.09 | 2246 | 60.6 |
| Lawsonibacter hominis ADS36 | Domain: Bacteria; phylum: Bacillota_A; class: Clostridia; order: Oscillospirales; family: Oscillospiraceae; genus: NSJ-51; species: NSJ-51 sp900549795 | 2.74 | 2760 | 61.9 |
| Thermoplasmata archaeon RFS1 | Domain: Archaea; phylum: Thermoplasmatota; class: Thermoplasmata; order: Methanomassiliicoccales; family: Methanomethylophilaceae; genus: UBA71; species: UBA71 sp015063125 | 1.46 | 1502 | 53.8 |
| Methanomicrobium mobile RFS2 | Domain: Archaea; phylum: Halobacteriota; class: Methanomicrobia; order: Methanomicrobiales; family: Methanomicrobiaceae; genus: Methanomicrobium; species: Methanomicrobium mobile | 1.81 | 1861 | 49.1 |
| Bacteroidales bacterium RFS3 | Domain: Bacteria; phylum: Bacteroidota; class: Bacteroidia; order: Bacteroidales; family: UBA932; genus: Egerieousia; species: Egerieousia sp900316795 | 2.36 | 2122 | 47.1 |
| Bacteroidales bacterium RFS4 | Domain: Bacteria; phylum: Bacteroidota; class: Bacteroidia; order: Bacteroidales; family: F082; genus: Limimorpha; species: Limimorpha sp017649945 | 2.38 | 2109 | 44.4 |
| Parabacteroides merdae RFS5 | Domain: Bacteria; phylum: Bacteroidota; class: Bacteroidia; order: Bacteroidales; family: Tannerellaceae; genus: Parabacteroides; species: Parabacteroides merdae | 4.41 | 3747 | 45.7 |
| Proteiniphilum acetatigenes RFS7 | Domain: Bacteria; phylum: Bacteroidota; class: Bacteroidia; order: Bacteroidales; family: Dysgonomonadaceae; genus: Proteiniphilum; species: Proteiniphilum acetatigenes | 4.81 | 4044 | 43.2 |
| Escherichia coli RFS8 | Domain: Bacteria; phylum: Pseudomonadota; class: Gammaproteobacteria; order: Enterobacterales; family: Enterobacteriaceae; genus: Escherichia; species: Escherichia coli | 4.76 | 4710 | 50.7 |
| Desulfovibrio vulgaris RFS9 | Domain: Bacteria; phylum: Desulfobacterota; class: Desulfovibrionia; order: Desulfovibrionales; family: Desulfovibrionaceae; genus: Nitratidesulfovibrio; species: Nitratidesulfovibrio vulgaris | 3.11 | 3075 | 63.4 |
| Pyramidobacter sp. RFS10 | Domain: Bacteria; phylum: Synergistota; class: Synergistia; order: Synergistales; family: Dethiosulfovibrionaceae; genus: Pyramidobacter; species: Pyramidobacter sp002007215 | 2.58 | 2684 | 60.3 |
| Synergistes jonesii RFS11 | Domain: Bacteria; phylum: Synergistota; class: Synergistia; order: Synergistales; family: Synergistaceae; genus: Synergistes; species: Synergistes jonesii | 2.46 | 2492 | 56.8 |
| Aminobacterium mobile RFS12 | Domain: Bacteria; phylum: Synergistota; class: Synergistia; order: Synergistales; family: Aminobacteriaceae; genus: Aminobacterium; species: Aminobacterium mobile | 2.06 | 2020 | 43.8 |
| Aminobacterium sp. RFS13 | Domain: Bacteria; phylum: Synergistota; class: Synergistia; order: Synergistales; family: Aminobacteriaceae; genus: Aminobacterium; species: Aminobacterium sp002432275 | 2.36 | 2323 | 42.1 |
| Propionibacterium ruminifibrarum RFS14 | Domain: Bacteria; phylum: Actinomycetota; class: Actinomycetia; order: Propionibacteriales; family: Propionibacteriaceae; genus: Propionibacterium; species: Propionibacterium ruminifibrarum | 2.75 | 2638 | 68.7 |
| Actinomycetaceae bacterium RFS15 | Domain: Bacteria; phylum: Actinomycetota; class: Actinomycetia; order: Actinomycetales; family: Actinomycetaceae; genus: Asp313; species: Asp313 sp015062745 | 2.55 | 2486 | 61.5 |
| Slackia heliotrinireducens RFS16 | Domain: Bacteria; phylum: Actinomycetota; class: Coriobacteriia; order: Coriobacteriales; family: Eggerthellaceae; genus: Slackia; species: Slackia heliotrinireducens | 2.78 | 2564 | 60.2 |
| Petrotoga sp. RFS19 | Domain: Bacteria; phylum: Thermotogota; class: Thermotogae; order: Petrotogales; family: Petrotogaceae; genus: UBA5851; species: UBA5851 sp002431635 | 2.76 | 2641 | 37.7 |
| Enterococcus hirae RFS20 | Domain: Bacteria; phylum: Bacillota; class: Bacilli; order: Lactobacillales; family: Enterococcaceae; genus: Enterococcus_B; species: Enterococcus_B hirae | 2.78 | 2647 | 36.8 |
| Kandleria vitulina RFS21 | Domain: Bacteria; phylum: Bacillota; class: Bacilli; order: Erysipelotrichales; family: Coprobacillaceae; genus: Kandleria; species: Kandleria vitulina | 1.98 | 2013 | 35.2 |
| Clostridiales bacterium RFS24 | Domain: Bacteria; phylum: Bacillota_A; class: Clostridia; order: Peptostreptococcales; family: Anaerovoracaceae; genus: RUG13615; species: RUG13615 sp024697655 | 2.00 | 1948 | 56.4 |
| Paraclostridium dentum RFS25 | Domain: Bacteria; phylum: Bacillota_A; class: Clostridia; order: Peptostreptococcales; family: Peptostreptococcaceae; genus: Paraclostridium; species: Paraclostridium dentum | 1.56 | 1522 | 29.0 |
| Clostridium sp. RFS26 | Domain: Bacteria; phylum: Bacillota_A; class: Clostridia; order: Tissierellales; family: Tepidimicrobiaceae; genus: Schnuerera; species: Schnuerera sp021655095 | 3.08 | 3128 | 31.6 |
| Sporanaerobacter acetigenes RFS27 | Domain: Bacteria; phylum: Bacillota_A; class: Clostridia; order: Tissierellales; family: Sporanaerobacteraceae; genus: Sporanaerobacter; species: Sporanaerobacter acetigenes | 2.93 | 3011 | 31.2 |
| Clostridium sulfidigenes RFS28 | Domain: Bacteria; phylum: Bacillota_A; class: Clostridia; order: Clostridiales; family: Clostridiaceae; genus: Clostridium_J; species: Clostridium_J sulfidigenes | 2.94 | 2853 | 30.0 |
| [Clostridium] aminophilum RFS29 | Domain: Bacteria; phylum: Bacillota_A; class: Clostridia; order: Lachnospirales; family: Lachnospiraceae; genus: Clostridium_R; species: Clostridium_R aminophilum_A | 2.86 | 2687 | 51.3 |
| Enterocloster aldenensis RFS30 | Domain: Bacteria; phylum: Bacillota_A; class: Clostridia; order: Lachnospirales; family: Lachnospiraceae; genus: Enterocloster; species: Enterocloster aldenensis | 5.46 | 5552 | 50.5 |
| Enterocloster clostridioformis RFS31 | Domain: Bacteria; phylum: Bacillota_A; class: Clostridia; order: Lachnospirales; family: Lachnospiraceae; genus: Enterocloster; species: Enterocloster clostridioformis | 4.42 | 4651 | 49.8 |
| Clostridia bacterium RFS32 | Domain: Bacteria; phylum: Bacillota_A; class: Clostridia; order: Oscillospirales; family: CAG-272; genus: UMGS1696; species: UMGS1696 sp017540285 | 3.49 | 3330 | 61.6 |
| Clostridia bacterium RFS33 | Domain: Bacteria; phylum: Bacillota_A; class: Clostridia; order: Oscillospirales; family: Butyricicoccaceae; genus: UBA4644; species: UBA4644 sp017394205 | 1.99 | 2079 | 59.1 |
| Flavonifractor plautii RFS35 | Domain: Bacteria; phylum: Bacillota_A; class: Clostridia; order: Oscillospirales; family: Oscillospiraceae; genus: Flavonifractor; species: Flavonifractor plautii | 3.46 | 3682 | 61.3 |

^a^Determined from RASTtk v.1.073^4^ via KBase^5^

^b^Determined from QUAST v.4.4^6^ via KBase^5^

**Table 3.** MAGs are predicted to encode CAZymes as determined by dbCAN2^7^ on KBase^5^. GHs are glycoside hydrolases; GTs are glycosyltransferases, CBMs are carbohydrate-binding modules; CEs are carbohydrate esterase, PLs are polysaccharide lyases, AAs are auxiliary activities.

| MAG label | # GHs | # GTs | # CBMs | # CEs | # PLs | # AAs | # Total CAZymes |
| --- | --- | --- | --- | --- | --- | --- | --- |
| Thermoproteota archaeon ADS1 | 14 | 35 | 0 | 0 | 0 | 1 | 50 |
| Methanococcoides sp. ADS2 | 4 | 12 | 0 | 0 | 0 | 1 | 17 |
| Anaerohalosphaeraceae bacterium ADS3 | 288 | 20 | 8 | 11 | 11 | 1 | 339 |
| Sphaerochaeta sp. ADS4 | 38 | 2 | 0 | 4 | 5 | 0 | 49 |
| Bacteroidales bacterium ADS5 | 213 | 21 | 6 | 6 | 17 | 0 | 263 |
| Bacteroides uniformis ADS6 | 265 | 21 | 4 | 7 | 8 | 0 | 305 |
| Macellibacteroides fermentans ADS7 | 199 | 14 | 3 | 5 | 3 | 0 | 224 |
| Dysgonomonadaceae bacterium ADS8 | 104 | 10 | 4 | 4 | 1 | 0 | 123 |
| Petrimonas sp. ADS9 | 176 | 16 | 8 | 12 | 0 | 0 | 212 |
| Deltaproteobacteria bacterium ADS10 | 37 | 42 | 0 | 3 | 1 | 2 | 85 |
| Desulfovibrionaceae bacterium ADS11 | 36 | 30 | 0 | 0 | 0 | 1 | 67 |
| Desulfovibrionaceae bacterium ADS12 | 12 | 34 | 0 | 1 | 0 | 0 | 47 |
| Desulfovibrio aminophilus ADS13 | 12 | 26 | 0 | 1 | 0 | 0 | 39 |
| Candidatus Cloacimonetes bacterium ADS14 | 11 | 17 | 0 | 1 | 0 | 1 | 30 |
| Pyramidobacter piscolens ADS15 | 2 | 18 | 0 | 1 | 0 | 0 | 21 |
| Aminomonas paucivorans ADS16 | 14 | 12 | 0 | 1 | 0 | 0 | 27 |
| Aminobacterium colombiense ADS17 | 8 | 9 | 0 | 1 | 0 | 0 | 18 |
| Enterococcus lactis ADS18 | 122 | 11 | 1 | 5 | 6 | 2 | 147 |
| Acholeplasmataceae bacterium ADS19 | 8 | 1 | 0 | 1 | 0 | 0 | 10 |
| Bacillota bacterium ADS20 | 2 | 4 | 0 | 3 | 0 | 0 | 9 |
| Tepidanaerobacter sp. ADS21 | 9 | 7 | 0 | 5 | 0 | 0 | 21 |
| Clostridiaceae bacterium ADS22 | 10 | 11 | 0 | 2 | 0 | 0 | 23 |
| Anaerovoracaceae bacterium ADS23 | 15 | 7 | 0 | 4 | 0 | 0 | 26 |
| Acetoanaerobium noterae ADS24 | 26 | 8 | 0 | 1 | 0 | 0 | 35 |
| Proteiniborus sp. ADS25 | 23 | 8 | 0 | 4 | 0 | 1 | 36 |
| Clostridium tertium ADS26 | 143 | 20 | 2 | 8 | 4 | 2 | 179 |
| Clostridium paraputrificum ADS27 | 131 | 16 | 3 | 5 | 0 | 1 | 156 |
| Anaerotignum sp. ADS28 | 13 | 7 | 0 | 0 | 0 | 0 | 20 |
| [Clostridium] symbiosum ADS29 | 43 | 9 | 0 | 4 | 0 | 0 | 56 |
| [Clostridium] asparagiforme ADS30 | 90 | 10 | 0 | 9 | 6 | 0 | 115 |
| [Clostridium] citroniae ADS31 | 137 | 12 | 0 | 5 | 3 | 0 | 157 |
| Hydrogenoanaerobacterium saccharovorans ADS32 | 6 | 5 | 0 | 2 | 0 | 0 | 13 |
| Clostridia bacterium ADS33 | 3 | 5 | 0 | 3 | 0 | 0 | 11 |
| Oscillibacter ruminantium ADS34 | 15 | 9 | 0 | 2 | 0 | 0 | 26 |
| Pseudoflavonifractor sp. ADS35 | 14 | 10 | 0 | 1 | 0 | 0 | 25 |
| Lawsonibacter hominis ADS36 | 74 | 11 | 3 | 3 | 0 | 0 | 91 |
| Thermoplasmata archaeon RFS1 | 0 | 3 | 0 | 0 | 0 | 0 | 3 |
| Methanomicrobium mobile RFS2 | 0 | 4 | 0 | 0 | 0 | 0 | 4 |
| Bacteroidales bacterium RFS3 | 15 | 11 | 0 | 5 | 0 | 0 | 31 |
| Bacteroidales bacterium RFS4 | 28 | 17 | 1 | 1 | 1 | 0 | 48 |
| Parabacteroides merdae RFS5 | 264 | 23 | 5 | 8 | 12 | 0 | 312 |
| Proteiniphilum sp. RFS6 | 309 | 19 | 10 | 17 | 9 | 1 | 365 |
| Proteiniphilum acetatigenes RFS7 | 344 | 20 | 7 | 13 | 9 | 0 | 393 |
| Escherichia coli RFS8 | 91 | 32 | 2 | 8 | 0 | 6 | 139 |
| Desulfovibrio vulgaris RFS9 | 12 | 20 | 0 | 2 | 0 | 0 | 34 |
| Pyramidobacter sp. RFS10 | 2 | 17 | 0 | 1 | 0 | 0 | 20 |
| Synergistes jonesii RFS11 | 8 | 14 | 0 | 1 | 0 | 0 | 23 |
| Aminobacterium mobile RFS12 | 8 | 11 | 0 | 1 | 0 | 0 | 20 |
| Aminobacterium sp. RFS13 | 8 | 13 | 0 | 1 | 0 | 1 | 23 |
| Propionibacterium ruminifibrarum RFS14 | 65 | 11 | 0 | 3 | 0 | 0 | 79 |
| Actinomycetaceae bacterium RFS15 | 87 | 12 | 0 | 2 | 0 | 0 | 101 |
| Slackia heliotrinireducens RFS16 | 16 | 8 | 0 | 0 | 0 | 0 | 24 |
| Atopobiaceae bacterium RFS17 | 95 | 6 | 0 | 2 | 0 | 0 | 103 |
| Atopobiaceae bacterium RFS18 | 113 | 12 | 0 | 5 | 4 | 0 | 134 |
| Petrotoga sp. RFS19 | 102 | 13 | 0 | 4 | 0 | 0 | 119 |
| Enterococcus hirae RFS20 | 107 | 18 | 1 | 2 | 2 | 3 | 133 |
| Kandleria vitulina RFS21 | 80 | 13 | 1 | 2 | 0 | 0 | 96 |
| Erysipelotrichaceae bacterium RFS22 | 84 | 13 | 1 | 3 | 0 | 0 | 101 |
| Anaerovoracaceae bacterium RFS23 | 100 | 9 | 0 | 0 | 0 | 0 | 109 |
| Clostridiales bacterium RFS24 | 5 | 2 | 0 | 0 | 1 | 0 | 8 |
| Paraclostridium dentum RFS25 | 23 | 7 | 0 | 3 | 2 | 0 | 35 |
| Clostridium sp. RFS26 | 23 | 5 | 0 | 4 | 0 | 1 | 33 |
| Sporanaerobacter acetigenes RFS27 | 11 | 7 | 0 | 5 | 0 | 0 | 23 |
| Clostridium sulfidigenes RFS28 | 5 | 11 | 0 | 4 | 0 | 0 | 20 |
| [Clostridium] aminophilum RFS29 | 19 | 9 | 0 | 2 | 0 | 0 | 30 |
| Enterocloster aldenensis RFS30 | 114 | 11 | 0 | 9 | 2 | 0 | 136 |
| Enterocloster clostridioformis RFS31 | 115 | 14 | 0 | 6 | 4 | 0 | 139 |
| Clostridia bacterium RFS32 | 193 | 7 | 1 | 6 | 4 | 0 | 211 |
| Clostridia bacterium RFS33 | 4 | 4 | 0 | 3 | 0 | 0 | 11 |
| Oscillospiraceae bacterium RFS34 | 48 | 10 | 0 | 2 | 0 | 0 | 60 |
| Flavonifractor plautii RFS35 | 18 | 9 | 1 | 4 | 0 | 0 | 32 |
| Bacilli bacterium RFSC1 | 37 | 3 | 0 | 0 | 0 | 0 | 40 |

**Table 4**. CheckM statistics for the MAGs.

| MAG name | Completeness | Contamination | Strain heterogeneity |
| --- | --- | --- | --- |
| Thermoproteota archaeon ADS1 | 97.2 | 4.67 | 0 |
| Methanococcoides sp. ADS2 | 90.07 | 0 | 0 |
| Anaerohalosphaeraceae bacterium ADS3 | 95.45 | 2.27 | 0 |
| Sphaerochaeta sp. ADS4 | 93.18 | 0.1 | 100 |
| Bacteroidales bacterium ADS5 | 93.51 | 4.08 | 45.45 |
| Bacteroides uniformis ADS6 | 90.57 | 1.61 | 66.67 |
| Macellibacteroides fermentans ADS7 | 94.83 | 0.9 | 75 |
| Dysgonomonadaceae bacterium ADS8 | 92.22 | 3.46 | 37.5 |
| Petrimonas sp. ADS9 | 99.27 | 0.55 | 0 |
| Deltaproteobacteria bacterium ADS10 | 98.41 | 0.81 | 0 |
| Desulfovibrionaceae bacterium ADS11 | 96.83 | 0 | 0 |
| Desulfovibrionaceae bacterium ADS12 | 95.71 | 3.55 | 25 |
| Desulfovibrio aminophilus ADS13 | 98.03 | 4.64 | 50 |
| Candidatus Cloacimonetes bacterium ADS14 | 98.9 | 2.2 | 0 |
| Pyramidobacter piscolens ADS15 | 100 | 0.31 | 0 |
| Aminomonas paucivorans ADS16 | 98.31 | 0 | 0 |
| Aminobacterium colombiense ADS17 | 90.45 | 0 | 0 |
| Enterococcus lactis ADS18 | 96.71 | 0.06 | 0 |
| Acholeplasmataceae bacterium ADS19 | 99.33 | 0 | 0 |
| Bacillota bacterium ADS20 | 91.18 | 4.67 | 35.29 |
| Tepidanaerobacter sp. ADS21 | 95.19 | 0.96 | 0 |
| Clostridiaceae bacterium ADS22 | 99.19 | 4.03 | 0 |
| Anaerovoracaceae bacterium ADS23* | 95.04 | 0.71 | 0 |
| Acetoanaerobium noterae ADS24 | 98.46 | 2.1 | 0 |
| Proteiniborus sp. ADS25 | 99.1 | 0 | 0 |
| Clostridium tertium ADS26 | 92.18 | 0.97 | 100 |
| Clostridium paraputrificum ADS27 | 97.58 | 0 | 0 |
| Anaerotignum sp. ADS28 | 96.64 | 0 | 0 |
| [Clostridium] symbiosum ADS29 | 94.32 | 0 | 0 |
| [Clostridium] asparagiforme ADS30 | 92.36 | 1.39 | 66.67 |
| [Clostridium] citroniae ADS31 | 96.34 | 1.48 | 33.33 |
| Hydrogenoanaerobacterium saccharovorans ADS32 | 93.62 | 2.85 | 100 |
| Clostridia bacterium ADS33 | 95.97 | 0.67 | 0 |
| Oscillibacter ruminantium ADS34 | 99.33 | 2.01 | 0 |
| Pseudoflavonifractor sp. ADS35 | 95.97 | 0.67 | 0 |
| Lawsonibacter hominis ADS36 | 91.28 | 1.34 | 0 |
| Thermoplasmata archaeon RFS1* | 95.97 | 3.23 | 50 |
| Methanomicrobium mobile RFS2 | 97.39 | 0.65 | 0 |
| Bacteroidales bacterium RFS3 | 93.95 | 0.24 | 100 |
| Bacteroidales bacterium RFS4 | 97.58 | 0 | 0 |
| Parabacteroides merdae RFS5 | 96.92 | 0.02 | 0 |
| Proteiniphilum sp. RFS6 | 100 | 0.29 | 50 |
| Proteiniphilum acetatigenes RFS7 | 99.45 | 1.11 | 33.33 |
| Escherichia coli RFS8 | 99.93 | 0.1 | 0 |
| Desulfovibrio vulgaris RFS9 | 96.75 | 0.89 | 50 |
| Pyramidobacter sp. RFS10 | 96.61 | 0 | 0 |
| Synergistes jonesii RFS11 | 100 | 0 | 0 |
| Aminobacterium mobile RFS12 | 100 | 0 | 0 |
| Aminobacterium sp. RFS13* | 98.31 | 0 | 0 |
| Propionibacterium ruminifibrarum RFS14 | 91.01 | 1.32 | 33.33 |
| Actinomycetaceae bacterium RFS15 | 96.36 | 0.42 | 0 |
| Slackia heliotrinireducens RFS16 | 100 | 0 | 0 |
| Atopobiaceae bacterium RFS17 | 95.85 | 3.23 | 9.09 |
| Atopobiaceae bacterium RFS18 | 95.16 | 1.76 | 25 |
| Petrotoga sp. RFS19* | 98.28 | 1.72 | 0 |
| Enterococcus hirae RFS20 | 99.25 | 3.46 | 69.23 |
| Kandleria vitulina RFS21 | 98.58 | 0 | 0 |
| Erysipelotrichaceae bacterium RFS22 | 94.61 | 2.83 | 0 |
| Anaerovoracaceae bacterium RFS23 | 97.52 | 2.6 | 50 |
| Clostridiales bacterium RFS24 | 95.15 | 0.21 | 50 |
| Paraclostridium dentum RFS25 | 90.21 | 0 | 0 |
| Clostridium sp. RFS26 | 99.3 | 0.7 | 0 |
| Sporanaerobacter acetigenes RFS27 | 98.6 | 2.53 | 83.33 |
| Clostridium sulfidigenes RFS28 | 94.1 | 1.38 | 50 |
| [Clostridium] aminophilum RFS29 | 98.73 | 0 | 0 |
| Enterocloster aldenensis RFS30 | 97.45 | 0 | 0 |
| Enterocloster clostridioformis RFS31 | 96.04 | 0.56 | 0 |
| Clostridia bacterium RFS32 | 93.3 | 0 | 0 |
| Clostridia bacterium RFS33 | 93.96 | 0.67 | 0 |
| Oscillospiraceae bacterium RFS34 | 94.46 | 0.67 | 0 |
| Flavonifractor plautii RFS35 | 99.11 | 0.13 | 0 |
| Bacilli bacterium RFSC1 | 98.88 | 0 | 0 |

Statistics calculated using CheckM v.1.1.3^8^

*CheckM calculations for these four MAGs were performed after GenBank removal of known foreign contaminations.

**Table 5**. Full names, including accession numbers for genome and MAG references used to make the phylogenetic tree in Figure 2 of the main text.

| **Microbial name with accession numbers** | **Name used in phylogenetic tree** |
| --- | --- |
| Clostridiales bacterium (GCA_024697655.1_ASM2469765v1) | Clostridiales bacterium |
| Tepidanaerobacter sp. EBM_38 (GCF_001896545.1_ASM189654v1) | Tepidanaerobacter sp. EBM_38 |
| Deltaproteobacteria bacterium (GCA_012517535.1_ASM1251753v1) | Deltaproteobacteria bacterium |
| Bacteroidales bacterium (GCA_017649945.1_ASM1764994v1) | Bacteroidales bacterium (GCA_017649945.1) |
| Clostridium sp. Cult1 (GCF_021655095.1_ASM2165509v1) | Clostridium sp. Cult1 |
| Acholeplasmataceae bacterium (GCA_012839485.1_ASM1283948v1) | Acholeplasmataceae bacterium |
| Bacteroidales bacterium (GCA_012517575.1_ASM1251757v1) | Bacteroidales bacterium (GCA_012517575.1) |
| Clostridia bacterium (GCA_017540285.1_ASM1754028v1) | Clostridia bacterium (GCA_017540285.1) |
| Bacteroidales bacterium UBA1410 (GCA_002305015.1_ASM230501v1) | Bacteroidales bacterium UBA1410 |
| Desulfovibrio sp. UBA4079 (GCA_002382645.1_ASM238264v1) | Desulfovibrio sp. UBA4079 |
| Bacteroides uniformis (GCF_025147485.1_ASM2514748v1) | Bacteroides uniformis |
| Actinomycetaceae bacterium (GCA_015062745.1_ASM1506274v1) | Actinomycetaceae bacterium |
| uncultured Erysipelotrichaceae bacterium (GCA_902763555.1) | uncultured Erysipelotrichaceae bacterium |
| Faecalitalea sp. UBA4312 (GCA_002394635.1_ASM239463v1) | Faecalitalea sp. UBA4312 |
| Enterococcus lactis (GCF_015751045.1_ASM1575104v1) | Enterococcus lactis |
| [Clostridium] asparagiforme DSM 15981 (GCF_025149125.1_ASM2514912v1) | [Clostridium] asparagiforme DSM 15981 |
| Petrotoga sp. UBA5851 (GCA_002431635.1_ASM243163v1) | Petrotoga sp. UBA5851 |
| Petrimonas sp. IBARAKI (GCA_002356435.2_ASM235643v2) | Petrimonas sp. IBARAKI |
| Clostridiaceae bacterium (GCA_003527145.1_ASM352714v1) | Clostridiaceae bacterium |
| Atopobiaceae bacterium UBA1367 (GCA_002305995.1_ASM230599v1) | Atopobiaceae bacterium UBA1367 |
| Enterocloster clostridioformis (GCF_020297485.1_ASM2029748v1) | Enterocloster clostridioformis |
| Pseudoflavonifractor sp. Marseille-P3106 (GCA_900169975.1_PRJEB20005) | Pseudoflavonifractor sp. Marseille-P3106 |
| uncultured Bacteroidales bacterium (GCA_900316795.1) | uncultured Bacteroidales bacterium |
| Clostridia bacterium (GCA_017394205.1_ASM1739420v1) | Clostridia bacterium (GCA_017394205.1) |
| Cloacimonetes bacterium UBA5835 (GCA_002432865.1_ASM243286v1) | Cloacimonetes bacterium UBA5835 |
| Sphaerochaeta sp. UBA5849 (GCA_002432715.1_ASM243271v1) | Sphaerochaeta sp. UBA5849 |
| Parabacteroides merdae ATCC 43184 (GCF_025151215.1_ASM2515121v1) | Parabacteroides merdae ATCC 43184 |
| Clostridiales bacterium UBA4139 (GCA_002380805.1_ASM238080v1) | Clostridiales bacterium UBA4139 |
| Aminobacterium sp. UBA5845 (GCA_002432275.1_ASM243227v1) | Aminobacterium sp. UBA5845 |
| Methanococcoides sp. EBM-47 (GCA_001896725.1_ASM189672v1) | Methanococcoides sp. EBM-47 |
| Thermoplasmata archaeon (GCA_015063125.1_ASM1506312v1) | Thermoplasmata archaeon |
| [Clostridium] citroniae WAL-17108 (GCF_000233455.1) | [Clostridium] citroniae WAL-17108 |
| [Clostridium] aminophilum (GCF_900101295.1) | [Clostridium] aminophilum |
| Enterococcus hirae ATCC 9790 (GCF_000271405.2) | Enterococcus hirae ATCC 9790 |
| Desulfovibrio vulgaris str. Hildenborough (GCF_000195755.1) | Desulfovibrio vulgaris str. Hildenborough |
| Escherichia coli DSM 30083 = JCM 1649 = ATCC 11775 (GCF_003697165.2) | Escherichia coli DSM 30083 |
| Macellibacteroides fermentans (GCF_013409575.1) | Macellibacteroides fermentans |
| Synergistes jonesii (GCF_000712295.1) | Synergistes jonesii |
| Proteiniphilum acetatigenes DSM 18083 (GCF_000380985.1) | Proteiniphilum acetatigenes DSM 18083 |
| Kandleria vitulina DSM 20405 (GCF_000702065.1) | Kandleria vitulina DSM 20405 |
| Oscillibacter ruminantium GH1 (GCF_000307265.1) | Oscillibacter ruminantium GH1 |
| Clostridium tertium (GCF_017873245.1) | Clostridium tertium |
| Clostridium paraputrificum (GCF_900447045.1) | Clostridium paraputrificum |
| Aminobacterium mobile DSM 12262 (GCF_000526395.1) | Aminobacterium mobile DSM 12262 |
| Aminobacterium colombiense DSM 12261 (GCF_000025885.1) | Aminobacterium colombiense DSM 12261 |
| Slackia heliotrinireducens DSM 20476 (GCF_000023885.1) | Slackia heliotrinireducens DSM 20476 |
| Acetoanaerobium noterae (GCF_900168025.1) | Acetoanaerobium noterae |
| [Clostridium] symbiosum ATCC 14940 (GCF_000466485.1) | [Clostridium] symbiosum ATCC 14940 |
| Flavonifractor plautii ATCC 29863 (GCF_000239295.1) | Flavonifractor plautii ATCC 29863 |
| Pyramidobacter sp. C12-8 (GCF_002007215.1) | Pyramidobacter sp. C12-8 |
| Aminomonas paucivorans DSM 12260 (GCF_000165795.1) | Aminomonas paucivorans DSM 12260 |
| Enterocloster aldenensis (GCF_003434055.1) | Enterocloster aldenensis |
| Lawsonibacter hominis (GCF_014287895.1) | Lawsonibacter hominis |
| Paraclostridium dentum (GCF_012922555.1) | Paraclostridium dentum |
| Propionibacterium ruminifibrarum (GCF_900289195.1) | Propionibacterium ruminifibrarum |
| Sporanaerobacter acetigenes DSM 13106 (GCF_900130025.1) | Sporanaerobacter acetigenes DSM 13106 |
| Hydrogenoanaerobacterium saccharovorans (GCF_016901815.1) | Hydrogenoanaerobacterium saccharovorans |
| Clostridium sulfidigenes (GCF_000732635.1) | Clostridium sulfidigenes |
| Desulfovibrio aminophilus DSM 12254 (GCF_000422565.1) | Desulfovibrio aminophilus DSM 12254 |
| Pyramidobacter piscolens W5455 (GCF_000177335.1) | Pyramidobacter piscolens W5455 |
| Methanomicrobium mobile BP (GCF_000711215.1) | Methanomicrobium mobile BP |

**Figure 1**. Microbial enrichments cultivated in the presence of plastics produce acetate as measured by high-performance liquid chromatography, thus indicating growth.

**Figure 2**. Measurements from the initial passage of the microbial enrichments show the accumulation of pressure, indicating growth. Enrichment labels are explained in Table 1, with the exception of control samples (those with a “C” on the end) and NS-R-PS which was inoculated from goat feces, contained no plastic in the media, and had penicillin and streptomycin added.

References

1. Camacho C, Coulouris G, Avagyan V, Ma N, Papadopoulos J, Bealer K, Madden TL. BLAST+: architecture and applications. *BMC Bioinformatics*. 2009;10:421. doi:10.1186/1471-2105-10-421

2. Mistry J, Chuguransky S, Williams L, Qureshi M, Salazar GA, Sonnhammer ELL, Tosatto SCE, Paladin L, Raj S, Richardson LJ, Finn RD, Bateman A. Pfam: The protein families database in 2021. *Nucleic Acids Res*. 2021;49(D1):D412-D419. doi:10.1093/nar/gkaa913

3. Zheng J, Ge Q, Yan Y, Zhang X, Huang L, Yin Y. dbCAN3: automated carbohydrate-active enzyme and substrate annotation. 2023;51:W115-W121.

4. Brettin T, Davis JJ, Disz T, Edwards RA, Gerdes S, Olsen GJ, Olson R, Overbeek R, Parrello B, Pusch GD, Shukla M, Thomason JA, Stevens R, Vonstein V, Wattam AR, Xia F. RASTtk: A modular and extensible implementation of the RAST algorithm for building custom annotation pipelines and annotating batches of genomes. *Sci Rep*. 2015;5:8365. doi:10.1038/srep08365

5. Arkin AP, Cottingham RW, Henry CS, Harris NL, Stevens RL, Maslov S, Dehal P, Ware D, Perez F, Canon S, Sneddon MW, Henderson ML, Riehl WJ, Murphy-Olson D, Chan SY, Kamimura RT, Kumari S, Drake MM, Brettin TS, Glass EM, Chivian D, Gunter D, Weston DJ, Allen BH, Baumohl J, Best AA, Bowen B, Brenner SE, Bun CC, Chandonia JM, Chia JM, Colasanti R, Conrad N, Davis JJ, Davison BH, Dejongh M, Devoid S, Dietrich E, Dubchak I, Edirisinghe JN, Fang G, Faria JP, Frybarger PM, Gerlach W, Gerstein M, Greiner A, Gurtowski J, Haun HL, He F, Jain R, Joachimiak MP, Keegan KP, Kondo S, Kumar V, Land ML, Meyer F, Mills M, Novichkov PS, Oh T, Olsen GJ, Olson R, Parrello B, Pasternak S, Pearson E, Poon SS, Price GA, Ramakrishnan S, Ranjan P, Ronald PC, Schatz MC, Seaver SMD, Shukla M, Sutormin RA, Syed MH, Thomason J, Tintle NL, Wang D, Xia F, Yoo H, Yoo S, Yu D. KBase: The United States Department of Energy Systems Biology Knowledgebase. *Nat Biotechnol*. 2018;36(7):566-569. doi:10.1038/nbt.4163

6. Gurevich A, Saveliev V, Vyahhi N, Tesler G. QUAST: Quality assessment tool for genome assemblies. *Bioinformatics*. 2013;29(8):1072-1075. doi:10.1093/bioinformatics/btt086

7. Zhang H, Yohe T, Huang L, Entwistle S, Wu P, Yang Z, Busk PK, Xu Y, Yin Y. dbCAN2: a meta server for automated carbohydrate-active enzyme annotation. *Nucleic Acids Res*. 2018;46(W1):W95-W101. doi:10.1093/nar/gky418

8. Parks DH, Imelfort M, Skennerton CT, Hugenholtz P, Tyson GW. CheckM: Assessing the quality of microbial genomes recovered from isolates, single cells, and metagenomes. *Genome Res*. 2015;25(7):1043-1055. doi:10.1101/gr.186072.114
